# Post-partum trend in blood pressure levels, renal function and proteinuria in women with severe preeclampsia and eclampsia in Sub-Saharan Africa: A 6-months cohort study

**DOI:** 10.1186/1471-2393-14-134

**Published:** 2014-04-09

**Authors:** Francois Folefack Kaze, Francis A Njukeng, Andre-Pascal Kengne, Gloria Ashuntantang, Robinson Mbu, Marie Patrice Halle, Tazoacha Asonganyi

**Affiliations:** 1 Department of Internal Medicine and Specialties, Faculty of Medicine and Biomedical Sciences & University Teaching Hospital of Yaoundé, University of Yaoundé 1, Yaoundé, Cameroon; 2 Faculty of Medicine and Biomedical Sciences, University of Yaoundé 1, Yaoundé, Cameroon; 3 South African Medical Research Council & University of Cape Town, Cape Town, South Africa; 4 Department of Internal Medicine and Specialties, Faculty of Medicine and Biomedical Sciences & Yaoundé General Hospital, University of Yaoundé 1, Yaoundé, Cameroon; 5 Department of Obstetrics and Gynecology, Faculty of Medicine and Biomedical Sciences & Yaoundé Central Maternity, University of Yaoundé 1, Yaoundé, Cameroon; 6 Department of Internal Medicine, Faculty of Medicine and Pharmaceutical Sciences & Douala General Hospital, University of Douala, Douala, Cameroon; 7 Department of Biochemistry and physiologic Sciences, Faculty of Medicine and Biomedical Sciences, University of Yaoundé 1, Yaoundé, Cameroon

**Keywords:** Preeclampsia, Eclampsia, Hypertension, Proteinuria

## Abstract

**Background:**

Preeclampsia and eclampsia, which are the most frequent hypertensive disorders in pregnancy, are associated with renal involvements. We aimed to assess the time trend in blood pressure levels, renal function and proteinuria after delivery, and investigate their determinants in Cameroonian women with severe preeclampsia and eclampsia.

**Methods:**

This was a prospective cohort study involving 54 women with severe preeclampsia and eclampsia, conducted between July 2010 and February 2012 at the central maternity unit of the Yaoundé Central Hospital. Clinical and laboratory parameters were recorded from day-1 to 6 months after delivery. Mixed-linear and logistic regression models were used to relate baseline and within follow-up levels of covariates, with changes in blood pressure levels, renal function and proteinuria, as well as persisting hypertension, renal failure and proteinuria.

**Results:**

During follow-up, a significant improvement was observed in blood pressure, renal function and proteinuria (all p < 0.002). Thirteen (24.1%) patients with renal failure at delivery recovered completely within six weeks. Twenty-six (48.1%), 17 (31.5%) and 1 (1.8%) patients had persisting proteinuria at 6 weeks, 3 months and 6 months post-delivery, respectively. Corresponding figures for persisting hypertension were 23 (42.6%), 15 (27.8%) and 8 (14.8%). Advanced age, higher body mass index, low gestational age at delivery, low fetal birth weight, and proteinuria at delivery were the main risk factors for persisting hypertension at 3 months, meanwhile low fetal birth weight, severe preeclampsia and proteinuria at delivery were correlated with persisting proteinuria at 3 months. Advanced age and higher body mass index were the only determinants of the composite outcome of persisting hypertension or proteinuria at three and six months.

**Conclusion:**

Hypertension and proteinuria are very common beyond the postpartum period in Cameroonian women with severe preeclampsia and eclampsia. Long-term follow-up of these women will help preventing and controlling related complications.

## Background

Hypertensive disorders in pregnancy are a leading cause of maternal and perinatal morbidity and mortality worldwide, accounting for up to 25% of maternal deaths [1,2]. Preeclampsia and eclampsia, the most frequent hypertensive disorders, complicate approximately 5% of all pregnancies and account for up about 17% of maternal deaths and 12% of perinatal mortality [3-6].

Preeclampsia and eclampsia are pregnancy specific conditions with varying clinical features and associated multiple organs involvement. Both account for a third of acute kidney injury occurring in advanced pregnancy, with high risks of progression to end-stage renal disease (ESRD) [7-12]. The renal involvement in preeclampsia and eclampsia comprises a glomerular endotheliosis which is characterized by proteinuria and renal failure. After delivery, hypertension, proteinuria and renal failure are expected to resolve progressively [13]. However, hypertension and proteinuria can persist for years after delivery, necessitating further investigations for possible underlying renal disease [13-17]. Meanwhile, renal failure highly correlates with blood pressure levels and renal function usually assumes the normal range within postpartum [18].

High prevalence of preeclampsia and eclampsia related complications and risk factors have been reported among African women [2,15,19]. However, follow-up of women after delivery has been less-than-optimal in most studies. Where the follow-up has been successfully conducted, the nephrological evaluation has received only little attention, despite the high burden of ESRD in Sub-Saharan Africa setting. Therefore, knowledge of the long-term outcomes of preeclampsia and eclampsia among African women remains very limited.

This cohort study was conducted to monitor the time-trend in blood pressure levels, renal function and proteinuria up to six months after delivery in African women with severe preeclampsia and eclampsia. Additionally, we investigated the factors associated with persisting hypertension, renal failure and/or proteinuria during follow-up.

## Methods

### Study setting

This was a prospective cohort study involving women with severe preeclampsia and eclampsia, conducted between July 2010 and February 2012 at the central maternity unit of the Yaoundé Central Hospital, Cameroon. This was part of a study on hypertensive disorders in pregnancy carried out in the central maternity unit during this study period. The central maternity unit of the Yaoundé Central Hospital is one of the referral center for obstetrical conditions for the Capital city of Cameroon (Yaoundé) and surrounding areas. On a monthly basis, it provides antenatal care to about 500 pregnant women and performs on average 300 deliveries. The staffs of this unit comprise 10 gynecologists/obstetricians and 61 nurses. It is also a teaching unit for specialist obstetricians and gynecologists in training, as well as undergraduate medical students of the Yaoundé School of medicine. This study was approved by the Cameroon National Ethics Committee. All participants or their next-of-kin provided written informed consent before enrollment in the study.

### Data collection

Included participants were pregnant women with severe preeclampsia or eclampsia, who had attended at least three antenatal visits, and who subsequently delivered at the central maternity unit of the Yaoundé Central Hospital. These visits had to have been accrued within the last three months preceding the pregnancy and before the 20^th^ week of gestation. We excluded from the study, women with a known history of hypertension, diabetes mellitus, kidney disease, or a positive antenatal serology tests for any of the following infections: HIV, viral hepatitis B and C, toxoplasmosis, syphilis, rubella, cytomegalovirus, herpes simplex virus, coxsackie virus, varicella-zoster virus and parvovirus B19. However, no renal biopsy was performed to exclude any underlying renal disease. Participants who were lost to follow-up before the sixth month after delivery were also excluded. For each eligible participants, we collected clinical and laboratory data from day-1 postpartum to 6 months after delivery. Clinical data included socio-demographic details (age and marital status), personal and familial history of preeclampsia or eclampsia, systolic and diastolic blood pressure, body mass index (BMI), pregnancy characteristics (gravidity, type of gestation and paternity, gestational age at diagnosis of preeclampsia or eclampsia and at delivery, diagnosis-to-delivery time interval, mode of delivery, antihypertensive treatment), fetal birth weight and outcome. Laboratory data included serum and urinary creatinine, and urinary protein. The methods for urine and blood specimen analysis at the Yaoundé Central Hospital’s laboratory have been described in details elsewhere [20]. Secondary variables were derived from primary variables using validated formulas. Estimated glomerular filtration rate (eGFR) was based on the Cockroft-Gault formula. Systolic and diastolic blood pressures were monitored daily during hospitalization, weekly for 6 weeks after discharge, then at 3 months and 6 months after delivery. Meanwhile serum creatinine and proteinuria (urine protein to creatinine ratio) were recorded at day-1, every week from week-2 to week-6, then at 3 months and 6 months after delivery. Additional measurements of serum creatinine and proteinuria could be done to assist the management of the patient at the discretion of the attending healthcare team. Such intermediate measurements are not included in the current study.

Severe preeclampsia was defined by the occurrence after 20 weeks of pregnancy of blood pressure levels (systolic/diastolic) of 160/110 mmHg or more, and/or proteinuria above 5000 mg per day (estimated from the urine protein to creatinine ratio). Eclampsia was defined by the onset of seizures in a participant with preeclampsia. Participants, who were first diagnosed with severe preeclampsia and subsequently convulsed, were assigned to the eclampsia group. Persisting hypertension was defined by the continuous use of antihypertensive drugs to maintain blood pressure levels below 140/90 mmHg. Persisting proteinuria was defined by proteinuria above 200 mg per day (estimated from the urine protein to creatinine ratio) [21]. Renal failure was defined by serum creatinine levels above 1.1 mg/dl and/or creatinine clearance less than 90 ml/min [22].

### Statistical analysis

Data were analyzed with the used of the SAS-STAT® software v.9.1 for Windows®. Means and standard deviations, median and 25^th^-75^th^ percentiles, and counts and percentages were used to express results. The Fisher exact test, Student t-test and Mann–Whitney U test were used to compare qualitative and quantitative variables. Mixed linear regression models were used to examine changes in blood pressure, proteinuria, serum creatinine and eGFR during follow-up while adjusting for baseline and changing levels of potential confounders during follow-up. Determinants of persisting hypertension, proteinuria or any of the two at 3 and 6 months of follow-up were investigated through logistic regression models. All models were adjusted for age at baseline, body mass index and systolic blood pressure at the diagnosis of severe preeclampsia or eclampsia. A p-value <0.05 was used to indicate statistically significant results.

## Results

### Characteristics of the study population

During the study period, 5610 women were received for antenatal care and 5765 deliveries performed. Hypertensive disorders were present in 569 (9.8%) pregnant women including 261 (45.9%) with gestational hypertension, 125 (21.9%) with chronic hypertension, 117 (20.6%) with preeclampsia and 66 (11.6%) with eclampsia. Among those with preeclampsia and eclampsia, 56 (30.6%) consulted after 20 weeks of pregnancy, 53 (28.9%) had superimposed preeclampsia/eclampsia and 15 (8.2%) had mild preeclampsia.

Therefore, a total of 59/569 (10.4%) pregnant women with severe preeclampsia and eclampsia, fulfilling our inclusion criteria were recruited during the study period. Two pregnant women were lost to follow-up and three died on day-8, day-12 and 10 weeks after delivery respectively for acute kidney injury (could not afford dialysis for financial reasons), pulmonary embolism and pulmonary edema. Our analytic sample therefore included 54 participants who were followed up for 6 months. They comprised 37 (68.5%) participants with severe preeclampsia and 17 (31.5%) with eclampsia. As presented in Table 1, participants with eclampsia were significantly younger, more likely to deliver prematurely (via emergency caesarean section), to have newborns with low birth weight, to have a higher frequency of still birth, and to be treated with parenteral antihypertensive drugs (all p < 0.028).

**Table 1:** Characteristics overall and by diagnostic categories

**Characteristics**	**Overall**	**Severe preeclampsia**	**Eclampsia**	**p**
n	54	37	17	
Age, years (SD)	26.3 (6.6)	27.6 (6.5)	23.3 (5.9)	0.025
Married (%)	23 (42.6)	17 (45.9)	6 (35.3)	0.56
Weight, kg (SD)	76.9 (14.6)	78.4 (14.4)	73.6 (14.8)	0.27
Body mass index, Kg/m^2^ (SD)	26.3 (4.8)	26.7 (4.7)	25.4 (5.2)	0.37
Systolic blood pressure, mmHg (SD)	167.2 (19.6)	166.6 (16.9)	168.7 (25.1)	0.71
Diastolic blood pressure, mmHg (SD)	104.1 (17.6)	104.6 (17.2)	102.9 (19.0)	0.75
Personal history, n (%)	9 (16.7)	9 (24.3)	0	0.044
Family history, n (%)	2 (3.7)	2 (5.4)	0	>0.99
Gravidity, median (min-max)	1 (1–3)	2 (1–4)	1 (1–1)	0.018
Para, median (min-max)	1 (0–2)	1 (1–2)	1 (0–1)	0.007
Premature, median (min-max)	0 (0–1)	0 (0–1)	0 (0–1)	0.72
Aborted, median (min-max)	0 (0–1)	0 (0–1)	0 (0–1)	0.88
Alive, median (min-max)	1 (1–2)	1 (1–3)	1 (1–1)	0.0005
Primipartenity, n (%)	37 (68.5)	22 (59.4)	15 (88.2)	0.06
Single gestation, n (%)	49 (90.1)	34 (9.9)	15 (88.2)	0.64
Gestational age at diagnosis, week (SD)	34.3 (6.0)	34.8 (5.8)	33.3 (6.5)	0.42
Gestational age at delivery, week (SD)	36.9 (3.5)	37.6 (2.8)	35.4 (4.2)	0.028
Delivery – diagnosis time, day (median, min-max)	0 (0–20)	0 (0–20)	0 (0–17)	0.18
Time to delivery from diagnosis >1 week, n (%)	14 (25.9)	11 (29.7)	3 (17.6)	0.51
Vaginal delivery, n (%)	31 (57.4)	20 (54)	11 (64.7)	0.56
Birth weight, g (SD)	2732 (691)	2938 (586)	2286 (708)	0.0008
Foetal outcome, n (%)				<0.0001
Death	10 (18.5)	2 (5.4)	8 (47)	
Alive	44 (81.5)	35 (94.6)	9 (53)	
Treatment received				
Methyl-dopa tablet, n (%)	47 (87)	31 (83.8)	16 (94.1)	0.41
Nicardipine injection, n (%)	35 (64.8)	19 (51.3)	16 (94.1)	0.002
Nicardipine tablet, n (%)	32 (59.2)	23 (62.2)	9 (52.9)	0.56
Labetalol tablet, n (%)	4 (7.4)	3 (8.1)	1 (5.9)	>0.99
Diazepam injection, n (%)	5 (9.2)	0	5 (29.4)	0.002
Clorazepam tablet, n (%)	10 (18.5)	4 (10.8)	6 (35.3)	0.06
MgSO4 injection, n (%)	54 (100)	37 (100)	17(100)	NA
Outcomes				
Persisting hypertension at M3, n (%)	15 (27.8)	11 (29.7)	4 (23.5)	0.75
Persisting hypertension at M6, n (%)	8 (14.8)	6 (16.2)	2 (11.8)	>0.99
Persisting proteinuria at M3, n (%)	17 (31.5)	8 (21.6)	9 (52.9)	0.03
Persisting proteinuria at M6, n (%)	1 (1.8)	1 (2.7)	0	>0.99
Persisting hypertension or proteinuria at M3, n (%)	26 (48.1)	16 (43.2)	10 (58.8)	0.38
Persisting hypertension or proteinuria at M6, n (%)	9 (16.7)	7 (18.9)	2 (11.8)	0.70

M – Month; SD – Standard deviation.

### Trajectories of blood pressure, glomerular filtration rate and proteinuria during follow-up and prevalence of persisting abnormal levels at 3 and 6 months

**Figure 1:**
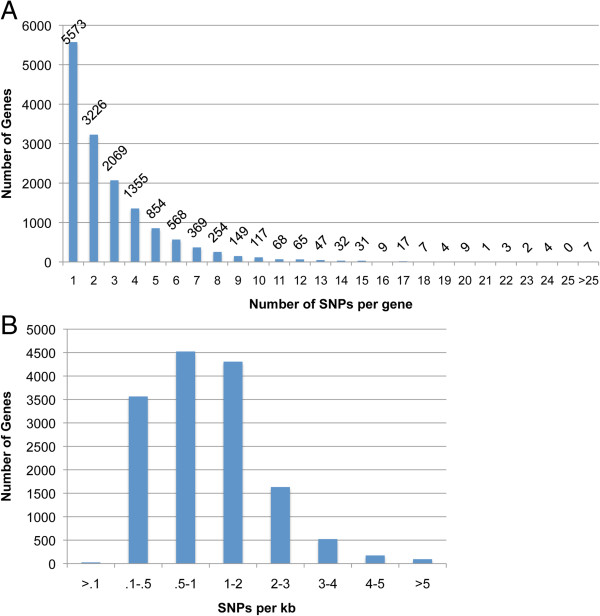
**Time trend in blood pressure and kidney function parameters during follow-up.** Figures are from mixed linear regression models with adjustment for baseline age, visit, diagnosis type and gestational age at delivery. Panels: **A** – Systolic and diastolic blood pressure; **B** – Serum creatinine; **C** – Estimated glomerular filtration rate; **D** – Estimated proteinuria.

The trajectories of key variables from mixed linear regressions models, adjusted for baseline age, type of hypertensive disorders and gestational age at delivery are depicted in Figure 1. Blood pressure levels and proteinuria significantly decreased while eGFR significantly increased across visits (all p ≤ 0.002). This pattern was similar in women with preeclampsia and those with eclampsia, with no evidence of statistical interaction (all p ≥ 0.11 for the interaction visit*type of hypertensive disorders); that is the trajectories of blood pressure levels and proteinuria across follow-up visits did not vary by type of hypertensive disorder.

Renal function assumed normal range in all women within three months after delivery while 13 (24.1%) and 10 (18.5%) patients had renal failure based on eGFR and serum creatinine respectively on day-1 post-partum. The prevalence of proteinuria decreased gradually with 26 (48.1%), 17 (31.5%) and 1 (1.8%) patients presenting persisting proteinuria at 6 weeks, 3 months and 6 months respectively. Similarly, hypertension prevalence decreased gradually with 23 (42.6%), 15 (27.8%) and 8 (14.8%) patients remaining hypertensive at 6 weeks, 3 months and 6 months respectively (Table 1).

### Determinants of hypertension and proteinuria

In age, systolic blood pressure and body mass index adjusted logistic regression models (Table 2), advanced age [odd ratio per one year 1.20 (95% confidence interval: 1.03-1.39)], and higher body mass index [1.20 (1.03-1.41) per kg/m^2^] were associated with increased risk of persisting hypertension at three months, meanwhile higher gestational age at delivery [0.71 (0.53-0.96) per week] and high fetal birth weight [0.41 (0.19-0.87) per 500 g higher] were associated with lower risk of persisting hypertension at three months. Higher fetal birth weight was also associated with lower risk of persisting proteinuria at 3 months [0.60 (0.37-0.98) per 500 g higher] while higher baseline urinary protein excretion was associated with increased risk of persisting proteinuria at 3 months [8.48 (1.36-52.79)], and with the risk of the composite outcome of persisting hypertension or proteinuria at 3 months [5.83 (1.43-23.84)]. Advanced age and higher BMI were the main determinants of the composite outcome of persisting hypertension or proteinuria at 6 months (Table 2).

**Table 2:** Determinants of three and six month’s outcomes of hypertension and proteinuria

**Variables**	**Persisting hypertension at 3 months**	**Persisting proteinuria at 3 months**	**Persisting hypertension or proteinuria at 3 months**	**Persisting hypertension or proteinuria at 6 months**
Age, per year	1.20 (1.03-1.39)	0.98 (0.89-1.09)	1.10 (0.99-1.22)	1.32 (1.03-1.70)
Marital status, single	1.20 (0.33-6.38)	0.69 (0.21-2.32)	0.53 (0.16-1.76)	1.24 (0.12-12.80)
BMI, per kg/m^2^	1.20 (1.03-1.41)	1.02 (0.80-1.17)	1.07 (0.93-1.23)	1.31 (1.07-1.60)
SBP baseline, per mmHg	0.98 (0.94-1.02)	1.01 (0.98-1.03)	0.99 (0.96-1.02)	1.01 (0.97-1.06)
DBP baseline, per mmHg	0.98 (0.91-1.05)	1.01 (0.96-1.06)	1.01 (0.96-1.06)	1.06 (0.93-1.20)
Severe pre-eclampsia, yes vs. no	0.42 (0.06-2.78)	0.20 (0.06-0.84)	0.22 (0.05-0.96)	0.99 (0.06-17.11)
Gravidity, per unit	1.01 (0.66-1.55)	0.78 (0.48-1.21)	1.05 (0.71-1.54)	1.30 (0.75-2.26)
Primipartenity, yes vs. no	0.64 (0.10-3.88)	0.83 (0.18-3.96)	1.10 (0.24-5.01)	0.88 (0.09-8.38)
Gestational age at delivery, per week	0.71 (0.53-0.96)	0.92 (0.77-1.09)	0.81 (0.66-0.99)	0.85 (0.62-1.15)
Time to delivery < 1 week, yes vs no	0.24 (0.03-1.75)	4.23 (0.67-30.47)	1.53 (0.31-7.51)	5.10 (0.27-97.86)
Route of delivery, CS	0.51 (0.10-2.61)	3.42 (0.92-12.64)	1.26 (0.37-4.25)	2.35 (0.21-25.79)
Birthweight (per 500 g)	0.41 (0.19-0.87)	0.60 (0.37-0.98)	0.49 (0.30-0.82)	0.92 (0.42-1.99)
Fetal outcome (alive)	3.05 (0.43-21.6)	1.57 (0.36-6.86)	2.42 (0.53-10.95)	0.46 (0.01-18.48)
Proteinuria at day 1, per unit	4.59 (1.27-16.55)	8.48 (1.36-52.79)	5.83 (1.43-23.84)	1.46 (0.30-6.80)
Creatinine at day 1, per unit	0.93 (0.15-5.87)	1.86 (0.62-5.54)	1.42 (0.42-4.76)	4.51 (0.45-45.31)

All models are adjusted for baseline age, SBP and BMI.

BMI – Body mass index; CS – Caesarean section; DBP – Diastolic blood pressure; SBP – Systolic blood pressure.

## Discussion

Our study has revealed in this group of women with pre-eclampsia and eclampsia, the high prevalence of persisting proteinuria and hypertension beyond the postpartum period and full recovery of renal function within the same period. Persisting hypertension and/or proteinuria correlated with increasing age and BMI, low gestational age at delivery, low fetal birth weight, and increased proteinuria at delivery, while baseline hypertensive disorder had no effect on the trajectories of blood pressure levels and proteinuria during follow-up. These results suggest that pregnant women with hypertensive disorders in this setting should be followed up for a much longer period after delivery, and where possible, investigations should be conducted to capture and adequately treat any underlying renal disease [6,8,11,12,16,17].

About a quarter of participants had acute kidney injury (acute renal failure) at delivery which complete resolved within the postpartum period as reported elsewhere [16,23]. This prevalence was lower than the 35.3% reported by Prakash et al. in India in their study on the spectrum of acute renal failure in late pregnancy [7]. Glomerular endotheliosis, which occurs in preeclampsia and eclampsia, decreases glomerular filtration rate and renal flow, and increases renal vascular resistance, predisposing patients to acute renal failure [6,24]. This renal involvement may persist for several years after delivery, indicating that women with pre-eclampsia or eclampsia should have extended nephrological follow-up.

The high prevalence of persisting hypertension observed among our study participants was associated with advanced age, increased BMI, low gestational age at delivery, low fetal birth weight and severity of proteinuria at delivery as reported elsewhere [15,23]. However, there have also been conflicting reports, with some suggesting a near-normalization of blood pressure levels within 4 weeks from delivery, and others observing much lower rates of hypertension at 6 weeks post-delivery [15,23]. During preeclampsia and eclampsia, generalized vasoconstriction occurs, leading to hypertension and increasing the long-term risk of hypertension from age-related systemic vascular stiffness with or without low initial nephron number [6,16,17,24]. In normal pregnancy, there are hemodynamic changes which activate the renin-angiotensin-aldosterone system, resulting in water and sodium retention, thus increase the BMI in later pregnancy [25]. Their association with generalized vasoconstriction during preeclampsia could explain the persistence of hypertension beyond the post-partum period.

We observed persisting near-complete resolution of proteinuria at six months post-partum as reported elsewhere [14,23]. Studies have reported an increase concentration of circulating soluble fms-like tyrosine kinase-1 in relation with glomerular endotheliosis and proteinuria. The later may persist many years after preeclampsia, in the form of micro- or macroalbuminuria, suggesting a slower resolution of pregnancy-related renal changes [6,16,17].

This study has some limitations including the absence of uric acid measurements, and liver evaluation to diagnose some complications like HELLP (hemolysis, elevated liver enzymes, low platelet) syndrome. Furthermore, our sample size may have affected our power for detecting some significant associations. Nevertheless, this study has the advantage of reporting, perhaps for the first time, the six month follow-up after delivery, of women with preeclampsia and eclampsia in the Sub-Saharan African setting without evidence of underlying renal disease according to the renal assessment done before pregnancy and 20 weeks of gestation. Furthermore, we took advantage of robust statistical methods to carefully investigate the time changes in key study parameters during follow-up, as well as the effects of their potential correlates.

## Conclusions

We have reported a high prevalence of hypertension and proteinuria beyond the postpartum period in women with pre-eclampsia and eclampsia in this setting. These patterns have been associated with increased future risk of chronic hypertension, ESRD and cardiovascular mortality [6,8,17]. Therefore, we suggest the need of an extended follow-up of these women beyond the postpartum period, particularly the older, those with high BMI, low gestational age at delivery, low fetal birth weight and increased proteinuria at delivery. This will allow the implementation of measures delay the onset and progression complications, as well as early detection, investigation and treatment of those who, in spite of preventive measures, will still develop those complications.

## Abbreviations

BMI: Body mass index; CS: Caesarean section; DBP: Diastolic blood pressure; EGFR: Estimated glomerular filtration rate; HTN: Hypertension; M: Month; PCR: Protein creatinine ratio; SBP: Systolic blood pressure.

## Competing interest

The authors report no conflicts of interest.

## Authors’ contribution

***Study conception ***– FFK, GA, RM, MPH. ***Clinical data collection and supervision ***– FAN, FFK, GA, RM. ***Acquisition and validation of the biological data ***– FFK, FAN, MPH, TA. ***Data analysis ***– APK. ***Data interpretation ***– FFK, APK, GA, TA. ***Manuscript drafting ***– FFK, FAN, APK. ***Critical revision of the manuscript ***– GA, MPH, RM, TA. ***Approval of the submission to the Journal ***– All coauthors.

## Pre-publication history

The pre-publication history for this paper can be accessed here:

http://www.biomedcentral.com/1471-2393/14/134/prepub
